# The Role of the Posterior Paraventricular Nucleus of the Thalamus in Food Deprivation‐Induced Heroin‐Seeking Relapse, in Male and Female Rats

**DOI:** 10.1111/adb.70115

**Published:** 2026-01-08

**Authors:** Catarina Borges, Anita Darecka, Amélie Mainville‐Berthiaume, Emily Ah‐Yen, Mahgol Darvishmolla, Richard Courtemanche, Uri Shalev

**Affiliations:** ^1^ Department of Psychology, Center for Studies in Behavioral Neurobiology Concordia University Montreal Quebec Canada; ^2^ Department of Health Kinesiology, and Applied Physiology, Center for Studies in Behavioral Neurobiology Concordia University Montreal Quebec Canada

**Keywords:** excitation, heroin, inhibition, pPVT, relapse, voluntary abstinence

## Abstract

Abstinence from drug use is often the result of the associated negative consequences. However, relapse occurs in a large proportion of abstinent users, and the underlying brain mechanisms are not clear. An arguably relevant brain area is the posterior paraventricular nucleus of the thalamus (pPVT), which plays a role in motivational processes and addiction‐like behaviours. Using a punishment‐imposed abstinence procedure, we assessed the effect of chemogenetic inhibition and excitation of the pPVT on food deprivation‐induced relapse to heroin‐seeking in male and female rats. Rats were trained to self‐administer heroin (0.1 mg/kg/infusion) for 2 weeks under a seeking–taking chain schedule. For punishment‐imposed abstinence, a mild footshock (0.2 to 0.6 mA) was delivered on 30% of the completed seek lever links instead of access to the take lever. Relapse to heroin‐seeking was tested after 24 h of food deprivation and under sated condition. Animals were injected (i.p.) with either a DREADD ligand or vehicle 15–20 min before the tests. There was no sex difference in heroin self‐administration or punishment‐imposed abstinence. Under the food deprivation condition, chemogenetic inhibition of the pPVT increased heroin‐seeking compared to the control group, only in male rats. Chemogenetic excitation of the pPVT resulted in an increase in heroin‐seeking under food deprivation conditions. Our results support an involvement of the pPVT in relapse to heroin‐seeking and suggest a sex‐dependent effect.

## Graphical abstract

Chemogenetic inhibition of the pPVT resulted in an increase in food‐deprivation‐induced heroin seeking compared to the control group, only in male rats. Chemogenetic excitation of the pPVT resulted in an increase in heroin seeking under food‐deprivation conditions. Our results suggest an involvement of the pPVT in acute food‐deprivation‐induced heroin seeking after punishment‐imposed abstinence.

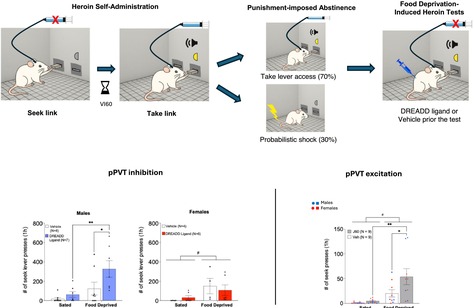

**Abbreviations**
ANOVA: analysis of variance
AP: anterior–posterior
CNO: Clozapine‐N‐oxide
DREADD: Designer Receptors Exclusively Activated by Designer Drugs
DV: dorsal‐ventral
FD: food deprivation
FR: fixed ratio
i.p.: intraperitoneal
ITI: inter‐trial interval
LFP: local field potential
ML: medial‐lateral
NAc: nucleus accumbens
pPVT: posterior paraventricular nucleus of the thalamus
PrL: prelimbic cortex
PVT: paraventricular nucleus of the thalamus
VI: variable interval

## 1 Introduction

The opioid epidemic has turned global [1], a clear example of the lack of efficient treatments to address opioid use disorder, including relapse to opioid use. Stress is one of the major triggers of relapse in humans and animal models [2, 3, 4, 5], and a great effort has been directed at elucidating the underlying brain mechanisms [6, 7]. We have previously reported a robust effect of acute food deprivation stress on the reinstatement of heroin‐seeking after extinction‐based abstinence [8, 9, 10], and after punishment‐imposed abstinence [11]. However, there are still considerable gaps in our understanding of the relevant neuronal circuits.

The paraventricular nucleus of the thalamus (PVT) is a mid‐thalamic region that has been receiving attention due to its central position in the brain and its contribution to diverse reward‐related behaviours [12]. However, the PVT has also been linked with responses to aversive stimuli [13]. Moreover, the PVT is necessary for the management of aversive and appetitive behaviours when those are in conflict [14, 15, 16]. Recent findings suggest that the PVT shows considerable heterogeneity in anatomical connection and function along the antero‐posterior axis [17]. The PVT is divided into anterior (aPVT) and posterior (pPVT) components, which differ in their anatomical and functional connectivity. In particular, the pPVT is highly associated with aversive states and regulation of stress‐induced behavioural responses [18], a function that is supported by the extensive projections of the pPVT to the extended amygdala [19]—a region linked to negative emotional behaviours [20]. For example, exposure to different acute stressors such as restraint and footshock increased c‐Fos mRNA in the pPVT, indicating increased neuronal activity [21, 22, 23, 24], and also activated pPVT‐nucleus accumbens (NAc) projections [25].

Considering its demonstrated role in the processing of emotionally relevant stimuli, it is not surprising that the PVT is involved in drug relapse across different types of drugs. Activation of the PVT has been observed during cue‐induced reinstatement of extinguished ethanol and cocaine‐seeking [26, 27]. Lesions of the PVT or its inactivation attenuated context‐induced reinstatement of alcohol‐seeking [28, 29] as well as cue and drug‐induced reinstatement of cocaine‐seeking [30, 31]. Chemogenetic inhibition that specifically targeted the PVT‐NAc pathway during retrieval of morphine conditioned place preference resulted in long‐term attenuation of morphine‐priming relapse [32]. Moreover, heroin self‐administration weakened synaptic efficacy in neurons of the PVT‐NAc pathway, and rescuing the activity in this pathway attenuated cue‐, priming‐ and stress‐induced reinstatement of heroin‐seeking [33].

More anatomically specifically, the pPVT is involved in drug relapse, with different triggers, abstinence types and substances. Inhibition of the prelimbic cortex (PrL)‐pPVT pathway attenuated context and cue‐induced reinstatement of cocaine‐seeking [34], and chemogenetic inhibition of the pPVT‐NAc pathway reduced cue‐induced relapse after forced abstinence [35]. In contrast, activation of the pPVT or the pPVT‐NAcShell pathway reduced heroin‐seeking only in chronically food‐restricted rats after forced abstinence [36, 37]. The contradicting findings regarding the role of the pPVT in relapse are puzzling and suggest a unique function of the pPVT under caloric challenge.

There is an ongoing effort to consider sex differences in all investigations of the brain mechanisms that underlie substance use disorder. The reports on potential sex‐specific effects in this context are quite mixed, and little is known about sex differences in the impact of stress on PVT function [38]. Nicolas et al. [39] found no sex differences in relapse/reinstatement to opioid‐seeking in rodent models. In contrast, recent opioid studies suggest that relapse vulnerability is higher in females than in males after extended fentanyl access [40] and after intermittent, but not continuous, heroin access. Sex‐specific effects for PVT function were demonstrated for chemogenetic inhibition of the PVT to the bed nucleus of the stria terminalis (BNST) projection, which resulted in binge alcohol drinking only in female mice [41]. These findings reinforce the importance of studying the potential sex‐specific involvement of the PVT in opioid relapse.

Here, we investigated the role of the pPVT in stress‐induced relapse to heroin‐seeking, after punishment‐imposed abstinence [11], in male and female rats. To that end, the pPVT was chemogenetically inhibited or activated prior to acute food deprivation‐induced heroin relapse tests in both sexes. Considering our previous findings with pPVT manipulations [36, 37], we hypothesized that inhibition of the pPVT would augment, while activation would attenuate food deprivation‐induced relapse to heroin‐seeking, after punishment‐imposed abstinence. Because of the lack of consistent existing data, we had no specific expectation for sex‐related differences.

## 2 Materials and Methods

Detailed information is provided in the Supporting Information.

### 2.1 Subjects

Male and female Long Evans rats (250–300 g on arrival; Charles River, QC) were housed on a reversed 12‐h light/dark cycle (lights off 9:30 AM) with ad libitum access to food (Teklad 2018C) and water. Rats were single‐housed postsurgery in operant chambers. All procedures were approved by the Concordia University Animal Research Ethics Committee and followed Canadian Council on Animal Care guidelines.

### 2.2 Apparatus

Operant chambers (Coulbourn Instruments; 29 × 29 × 25.5 cm) were housed in sound‐attenuating boxes and equipped with two retractable levers, a house light, cue lights, a tone generator (2.9 kHz) and an infusion pump.

### 2.3 Surgeries

Rats were implanted with intravenous catheters in the right jugular vein under isoflurane (2%) anaesthesia as previously described [37]. During the same surgery, 0.6 μL of viral vector was injected into the posterior paraventricular thalamus (pPVT; −3.0 AP, 1.15 ML, −5.6 DV, 12° angle) at 0.1 μL/min. Post‐op care included saline (2.0 mL, s.c.) and Ketoprofen (5.0 mg/kg, s.c.) for 3 days. *Viral Vectors for Designer Receptors Exclusively Activated by Designer Drugs* (DREADD; Canadian Neurophotonic Platform/Viral Vector Core, Québec, QC): Exp 1: AAV8‐hSyn‐hM4D(Gi)‐mCherry (inhibitory DREADD); Exp 2: AAV8‐hSyn‐hM3D(Gq)‐mCherry (excitatory DREADD); Exp 3: AAV8‐hSyn‐mCherry (only fluorophore); Exp 4: AAV8‐hSyn‐hM3D(Gq)‐mCherry (no catheterization).

### 2.4 Drugs

Heroin HCl (National Institute for Drug Abuse, Research Triangle Park, NC, USA): 0.1 mg/kg/infusion in sterile saline. For DREADD ligands, half of the rats in Experiment 1 were injected (i.p.) with Clozapine‐N‐oxide (CNO, National Institute for Drug Abuse, Research Triangle Park or Cayman Chemical, Ann Arbor, MI, USA), at 6.0 mg/kg in 5% DMSO + 95% saline. The other half of the rats in Experiment 1 and all rats in the other experiments were injected (i.p.) with JHU37160 (J60; HelloBio, Princeton, NJ, USA), at 0.1 mg/kg in saline.

### 2.5 Procedures

**FIGURE 1:**
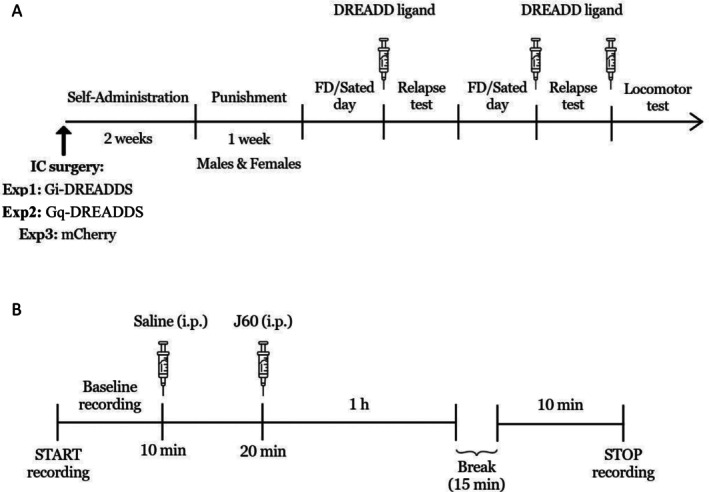
Timeline charts of the experimental design. FD: food deprivation (A) Experiments 1, 2 and 3. Excitation and inhibition of the pPVT. Syringes represent i.p. injections of the DREADD ligand (CNO: 0.6 mg/kg or JHU37160: 0.1 mg/kg) or vehicle, 15–20 min prior to the relapse or locomotor activity tests. (B) Experiment 4. In vivo electrophysiology validation of excitatory DREADDs. Syringes represent i.p. injections of JHU37160 (J60; 0.1 mg/kg) or saline.

A timeline for all experiments is provided in Figure 1.

#### 2.5.1 Heroin Self‐Administration

Rats self‐administered heroin (6 h/day) under a seek–take chain schedule, as previously described [42]. Briefly, training began with access only to the take lever (3 days). Take lever presses delivered a heroin infusion (0.13 mL over 5 s) paired with a 20‐s light‐tone cue (fixed ratio 1, FR1). In the following days, the seek lever was extended first. The first press on the seek lever initiated a variable interval (VI) schedule, and the first lever press after the expiry of the VI resulted in the retraction of the seek lever and insertion of the take lever. Take lever presses (FR1) delivered a heroin infusion as above. Seek lever schedules of reinforcement advanced over the training days: FR1 (4 days), VI5 (2–3 days), VI30 (3 days) and VI60 (3 days). Inter‐trial intervals (ITIs) increased from 30 s to 5 min over the days.

#### 2.5.2 Punishment Phase

For 6 days, 30% of completed seeking responses were followed by a 0.5‐s footshock (increasing daily by 0.1 mA from 0.2 to 0.6 mA) instead of taking lever access. The remaining 70% continued as during training. In Experiment 1, male rats received CNO, J60 or vehicle before the sessions to assess the effect of pPVT inhibition on behaviour under punishment. No behavioural effects were observed, and data are not reported here.

#### 2.5.3 Relapse Tests

Rats underwent two 1‐h heroin‐seeking tests (24‐h food deprived and sated, counterbalanced). No heroin or shock was delivered. The seek‐take schedule (VI60‐FR1, 5‐min ITI) was used. J60, CNO or vehicle was administered 20 min before each test session. Water was always available; food was returned immediately post‐test.

#### 2.5.4 Locomotor Activity

Rats were injected with J60 (0.1 mg/kg), CNO (6.0 mg/kg) or vehicle, and 20 min later, placed in activity chambers (Coulbourn Instruments) for 1 h. Total distance travelled was recorded (TruScan software).

#### 2.5.5 Electrophysiology

One rat was infused with AAV8‐hSyn‐hM3D(Gq)‐mCherry in the pPVT. After 8 weeks, neuronal activity was recorded under isoflurane, before and after J60 injection (0.1 mg/kg, i.p.), with saline injection as a control. Tungsten electrodes (3–4; 75 μm, 0.2–1.5 MΩ) were independently lowered into the pPVT (−3.0 AP, 0 ML, −5.4 DV). Signals were digitized (32 kHz), and spikes were sorted and analysed using SpikeSort and Neuralynx software. An electrolytic lesion (100 μA, 35 s) was made post‐recording.

#### 2.5.6 Histology

After final testing, rats were perfused with PBS, followed by 4% paraformaldehyde. Brains were postfixed (24 h), cryoprotected in 30% sucrose (48 h) and sectioned at 40 μm. The expression of the fluorophore mCherry and electrode placements was verified via fluorescence microscopy, with reference to the brain atlas [43].

### 2.6 Statistical Analyses

All statistical analyses were conducted using GraphPad Prism 9 or 10.2.3 (Boston, MA, USA).

#### 2.6.1 Behaviour

Self‐administration and punishment data were analysed using two‐way ANOVAs or mixed‐effects models with *Sex* as a between‐subject factor and *Training/punishment day* as a within‐subject factor. Because our design was not powered for comparing performance in the relapse tests between sexes, seek and take lever responses tests were analysed using two‐way ANOVAs, with *Treatment* (J60, vehicle) as the between‐subject factor and *feeding condition* (sated, food deprivation) as the within‐subject factor, collapsed over sex. Specific multiple comparisons were done using Bonferroni corrections. Locomotor activity data were analysed with an unpaired *t*‐test comparing J60 and vehicle groups. The effect sizes reported are Cohen's coefficient (*d*) and partial eta squared (*ƞ* ^*2*^). Statistical significance was defined as *p* ≤ 0.05.

#### 2.6.2 Electrophysiology

Multi‐ and single‐unit quantitative analyses were performed using NeuroExplorer (Nex Technologies, Littleton, MA) and MATLAB (MathWorks, Natick, MA). For unit data, the digitized spikes were processed for single‐unit identification. For single‐electrode recordings, this was done in SpikeSort (Neuralynx, Bozeman, MT, USA). Spike sorting excluded ISIs < 1 ms, while units with less than 2% ISIs under 3 ms were included. Data were binned (1 and 10 min) for firing rate analysis. One‐way ANOVA was performed on the firing rate data over time.

## 3 Results

### 3.1 Experiment 1: pPVT Inhibition During Relapse Test

#### 3.1.1 Self‐Administration Training

**FIGURE 2:**
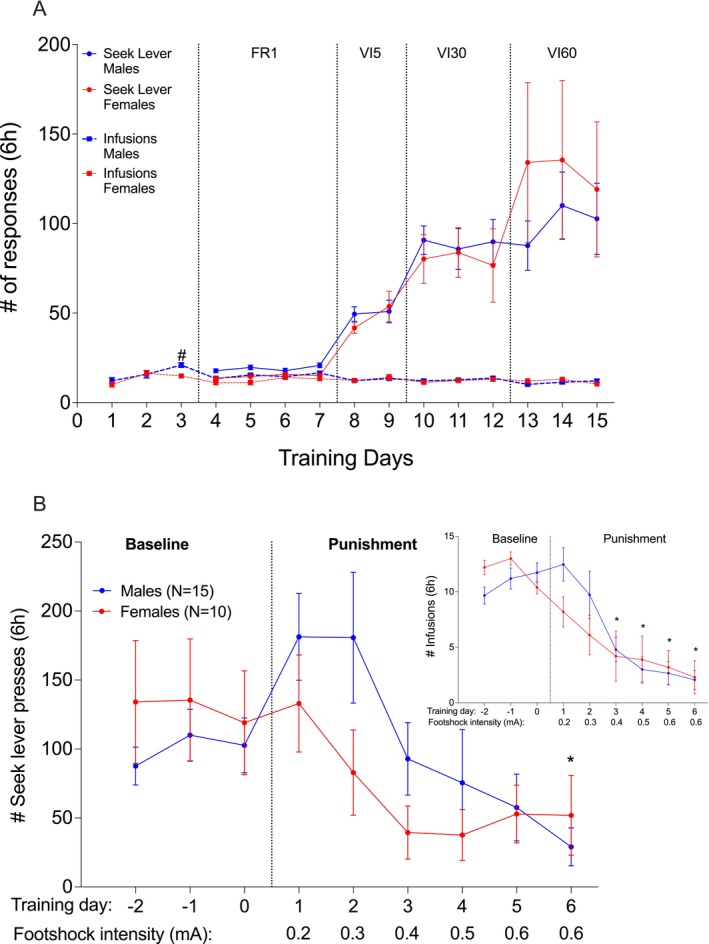
Experiment 1: pPVT inhibition. (A) Heroin self‐administration. Mean (±SEM) of seek lever presses (circles) and number of heroin infusions (0.1 mg/kg/infusion; squares) over training days for females (*N* = 10; red) and males (*N* = 15; blue). FR: fixed ratio, VI: variable interval schedule of reinforcement on the seek lever. # *p* = 0.001 males vs. females, Bonferroni‐corrected multiple comparisons. (B) Punishment‐imposed abstinence. Mean (±SEM) of seek lever responses and infusions (insert) over punishment days for females (red) and males (blue). Baseline is the last 3 days of training under VI60. *p < 0.05 compared to last baseline day, Bonferroni‐corrected multiple comparisons, collapsed over sex.

Ten female and five male rats were removed from the analysis due to health issues, failure to acquire heroin self‐administration training (< 3 infusions/session averaged across the last 3 days of training), or misplacement of the injector. Thus, 10 females and 15 males were included in the analyses. A slight decrease in take lever responses was observed compared to the take‐lever‐only days (*Training days*: *F*_(14, 318)_ = 7.038, *p* < 0.0001, *η* ^*2*^ = 0.237; Figure 2A). Seek lever responses increased over the training days (*Training days*: *F*_(11, 249)_ = 19.46, *p* < 0.0001, *η* ^*2*^ = 0.462). There were no overall statistically significant sex differences in the number of infusions (*Sex*: *F*_(1, 23)_ = 2.593, *p* = 0.121, *η* ^*2*^ = 0.101). The *Sex x Training days* interaction was significant (*F*_(14, 318)_ = 2.483, *p* = 0.002, *η* ^*2*^ = 0.099), and Bonferroni‐corrected multiple comparisons revealed a higher infusion number for the male rats on Day 3 of training (*t*_(341_) = 4.042, *p* = 0.001). There were no statistically significant sex differences in the number of seek lever presses (*Sex*: *F*_(1, 23)_ = 0.062, *p* = 0.806, *η* ^*2*^ = 0.003; *Sex x Training days: F*_(11, 249)_ = 0.888, *p* = 0.552, *η* ^*2*^ = 0.038).

#### 3.1.2 Punishment Phase

Both sexes decreased the number of seek lever presses (Figure 2B; *Punishment days*: *F*_(8, 183)_ = 5.135, *p* < 0.0001, *η* ^*2*^ = 0.183) and infusions (*Punishment days*: *F*_(8, 183)_ = 23.100, *p* < 0.0001, *η* ^*2*^ = 0.502) with the increase of footshock intensity. Post hoc tests are presented in Figure 2B. There was no statistically significant effect of *Sex* on the number of infusions (*F*_(1, 23)_ = 0.159, *p* = 0.694, *η* ^*2*^ = 0.007) or on the seek lever presses (*F*_(1, 23)_ = 0.226, *p* = 0.638, *η* ^*2*^ = 0.010). There were also no statistically significant *Sex x Punishment days* interaction effects for heroin infusions (*F*_(8, 183)_ = 1.913, *p* = 0.061, *η* ^*2*^ = 0.077) or seek lever presses (*F*_(8, 183)_ = 1.742, *p* = 0.092, *η* ^*2*^ = 0.071). A visible, but not statistically significant, increase in the number of seek lever presses and infusions was recorded at the beginning of the punishment phase. We have observed such increases before and suggested that they occur in response to the overall reduced access to the take lever with the introduction of punishment trials [11].

#### 3.1.3 Heroin‐Seeking Test

**FIGURE 3:**
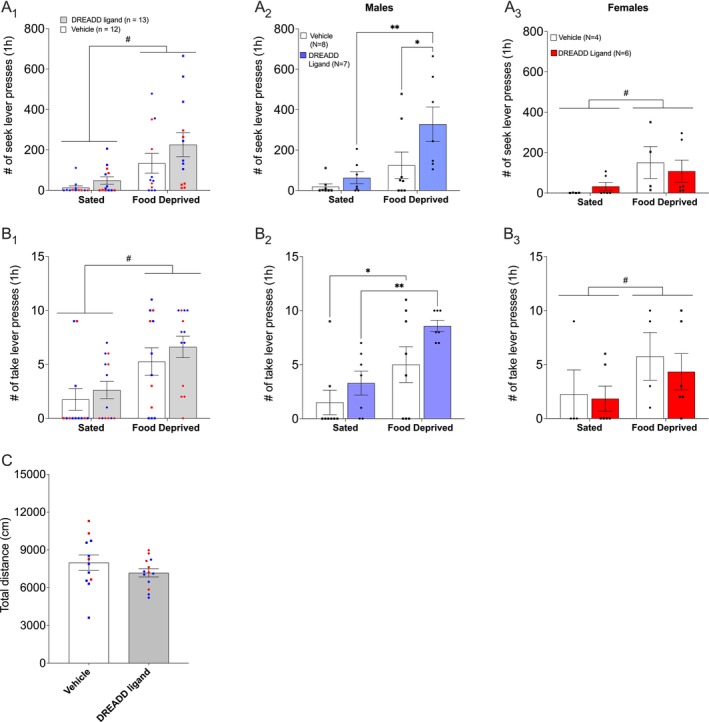
Experiment 1: pPVT inhibition. Food deprivation‐induced heroin‐seeking tests in male and female rats. Rats transduced with AAV8‐hSyn‐hM3D(Gi)‐mCherry were injected systemically (i.p.) with CNO (0.6 mg/kg) or JHU37160 (0.1 mg/kg) or vehicle, 15–20 min before the relapse test. Mean (±SEM) of seek lever responses (A_1_) and take lever responses (B_1_) during the 1‐h heroin relapse tests under food deprivation and sated conditions, combined for female (red) and male (blue) rats. # Two‐way ANOVA, food deprived vs. sated, *p* ≤ 0.0001. A_2_ and B_2_: mean (±SEM) number of seek lever responses and take lever responses, respectively, for male rats. *p < 0.04, **p < 0.003, Bonferroni‐corrected multiple comparisons. A_3_ and B_3_: mean (±SEM) number of seek lever responses and take lever responses, respectively, for female rats. * Two‐way ANOVA, food deprived vs. sated, *p* ≤ 0.02. (C) Locomotor activity test under sated conditions. Mean (±SEM) distance travelled (cm) in an open field for 1 h in rats with pPVT inhibited (DREADD ligand) and control rats (vehicle).

Food deprivation statistically significantly increased heroin‐seeking (*Feeding condition*: *F*_(1, 23)_ = 20.63, *p* = 0.0001, *η* ^*2*^ = 0.473; Figure 3A_1_). Inhibition of the pPVT did not result in a statistically significant effect on seek lever responses (*Treatment*: *F*_(1, 23)_ = 1.889, *p* = 0.182, *η* ^*2*^ = 0.076), and the *Treatment x Feeding* interaction was not statistically significant (*F*_(1, 23)_ = 0.750, *p* = 0.396, *η* ^*2*^ = 0.032). The same pattern was observed for the number of take lever responses (*Feeding condition*: *F*_(1, 23)_ = 36.53, *p* < 0.0001, *η* ^*2*^ = 0.614; *Treatment*: *F*_(1, 23)_ = 0.726, *p* = 0.403, *η* ^*2*^ = 0.031; *Treatment x Feeding*: *F*_(1, 23)_ = 0.162, *p* = 0.691, *η* ^*2*^ = 0.007; Figure 3B_1_).

Although the study design was not powered for sex comparisons, a closer look at the individual subjects' data suggested a robust effect of pPVT inhibition on heroin‐seeking in the male rats. We therefore compared the impact of pPVT inhibition under food deprivation and sated conditions separately in male and female rats using Bonferroni's multiple comparisons correction. In males, the food deprivation‐induced increase in seek lever presses in vehicle‐treated rats did not reach statistical significance (*t*_(13)_ = 1.736, *p* = 0.212, Cohen's *d* = 0.481). In contrast, food‐deprived rats that were treated with DREADD ligand showed a statistically significant increase in seek lever responses compared to the sated condition (*t*_(13)_ = 4.067, *p* = 0.003, Cohen's *d* = 1.128) and to the vehicle‐treated, food‐deprived rats (*t*_(26)_ = 2.589, *p* = 0.031, Cohen's *d* = 1.015; Figure 3A_2_). Exposure to food deprivation induced a statistically significant increase in take lever responses in both the vehicle and DREADD ligand‐treated rats (*p* = 0.02, Cohen's *d* = 0.838; *p* = 0.002, Cohen's *d* = 1.184, respectively). There was an increase in take lever presses in pPVT‐inhibited male rats, but no statistically significant differences between the treatment groups within the food‐deprived or sated conditions were found (*p* = 0.621, Cohen's *d* = 0.406; *p* = 0.100, Cohen's *d* = 0.812, respectively; Figure 3B_2_).

None of the Bonferroni‐corrected multiple comparisons of seek or take lever responses in the female rats was statistically significant (all *p*'s > 0.06), although overall, food deprivation increased seek lever pressing (*F*_(1, 8)_ = 8.863, *p* = 0.018, *η* ^*2*^ = 0.526; Figure 3A_3_) and take lever pressing (*F*_(1, 8)_ = 12.23, *p* = 0.008, *η* ^*2*^ = 0.605; Figure 3B_3_).

There were no statistically significant differences in locomotor activity between the vehicle and DREADD ligand groups in the open field (*t*_(23)_ = 1.203, *p* = 0.241, Cohen's *d* = 0.502).

### 3.2 Experiment 2: pPVT Excitation During Relapse Test

#### 3.2.1 Self‐Administration Training

**FIGURE 4:**
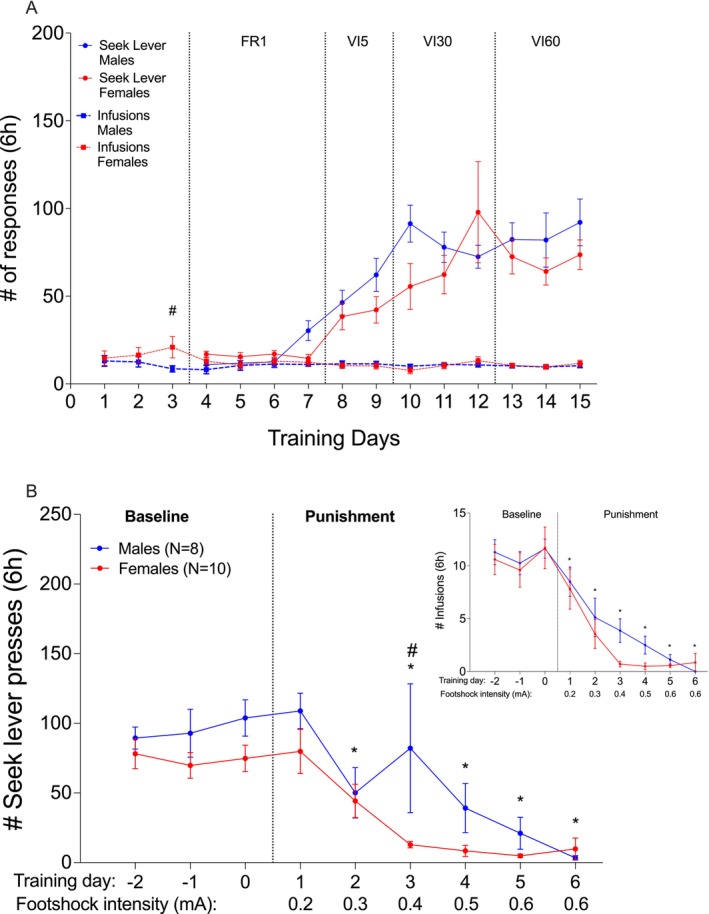
Experiment 2: pPVT activation. (A) Heroin self‐administration. Mean (±SEM) number of seek lever presses (circles) and number of heroin infusions (0.1 mg/kg/infusion; squares) over training days for females (*N* = 10; red) and males (*N* = 8; blue). FR: fixed ratio, VI: variable interval schedule of reinforcement on the seek lever. # *p* = 0.02 males vs. females, Bonferroni‐corrected multiple comparisons. (B) Punishment‐imposed abstinence. Mean (±SEM) of seek lever responses and infusions (insert) over punishment days for females (red) and males (blue). Baseline is the last 3 days of training under VI60. # *p* = 0.01 males vs. females; *p < 0.02, Bonferroni‐corrected multiple comparisons, compared to last baseline day, collapsed over sex.

Ten female and 12 male rats were removed from the analysis due to health issues, failure to acquire heroin self‐administration training or misplacement of the injector. Thus, 10 females and eight males were used in the analyses. Both sexes demonstrated an increase in seek lever presses as the VI increased over the training days (*Training days: F*_(11, 170)_ = 13.88, *p* < 0.0001, *η* ^*2*^ = 0.473) and a consistent number of heroin infusions over training days (*Training days: F*_(14, 222)_ = 1.381, *p* = 0.164, *η* ^*2*^ = 0.080, Figure 4A). The overall number of infusions received by each of the two sexes was not statistically significantly different (*Sex*: *F*_(1, 16)_ = 0.749, *p* = 0.400, *η* ^*2*^ = 0.045); neither were the seek lever presses (*Sex*: *F*_(1, 16)_ = 0.016, *p* = 0.901, *η* ^*2*^ = 0.0009). There was no statistically significant difference between sexes in the number of seek or take lever presses throughout the self‐administration period (*Sex x Training days: F*_(11, 170)_ = 1.061, *p* = 0.396, *η* ^*2*^ = 0.064; *F*_(14, 222)_ = 1.324, *p* = 0.195, *η* ^*2*^ = 0.077, respectively). Bonferroni‐corrected multiple comparisons revealed a higher infusion number for the male rats on Day 3 of training (*t*_(238)_ = 3.293, *p* = 0.017, Cohen's *d* = 0.427) compared to female rats.

#### 3.2.2 Punishment Phase

Both sexes decreased the number of seek lever presses (*Punishment days*: *F*_(8, 122)_ = 12.40, *p* < 0.0001, *η* ^*2*^ = 0.448; Figure 4B) and infusions (*Punishment days*: *F*_(8, 122)_ = 35.56, *p* < 0.0001, *η* ^*2*^ = 0.700) over the 6 punishment days. Bonferroni‐corrected multiple comparisons revealed a statistically significant lower number of infusions beginning on Day 1 (0.2 mA), and a lower number of seek lever presses beginning on Day 2 (0.3 mA), compared to the last baseline day. As in Experiment 1, although visual inspection suggested faster response inhibition in female rats, there was no overall statistically significant *Sex* effect or *Sex x Punishment days* interaction for heroin seek lever presses (*F*_(1, 16)_ = 4.331, *p* = 0.054, *η* ^*2*^ = 0.213; *F*_(8, 122)_ = 1.262, *p* = 0.270, *η* ^*2*^ = 0.076, respectively) or heroin‐taking (*F*_(1, 16)_ = 0.746, *p* = 0.401, *η* ^*2*^ = 0.045; *F*_(8, 122)_ = 0.596, *p* = 0.779, *η* ^*2*^ = 0.038, respectively). However, on the 0.4 mA day, female rats had a statistically significant lower number of seek lever presses compared to the males (Bonferroni‐corrected multiple comparisons: *t*_(138)_ = 3.335, *p* = 0.01, Cohen's *d* = 0.568).

#### 3.2.3 Heroin‐Seeking Test

**FIGURE 5:**
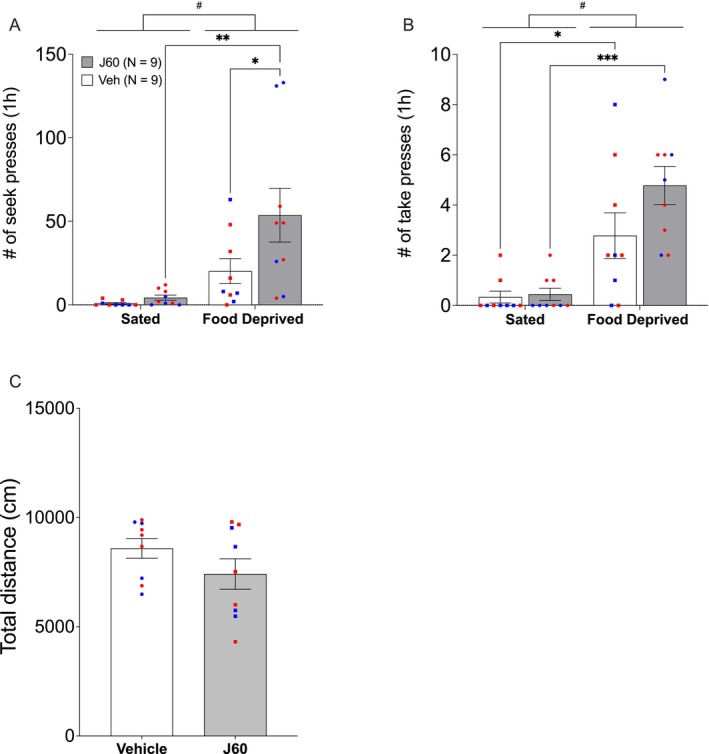
Experiment 2: pPVT activation. Food deprivation‐induced heroin‐seeking tests in male and female rats. Rats transduced with AAV8‐hSyn‐hM3D(Gq)‐mCherry were injected systemically (i.p.) with JHU37160 (J60; 0.1 mg/kg) or vehicle (Veh), 15–20 min before the relapse test. (A) Mean (±SEM) of seek lever responses during the 1‐h heroin relapse tests under food deprivation (FD) and sated conditions, for both female (red) and male (blue) rats. #Two‐way ANOVA, food deprived vs. sated, *p* < 0.002; *p = 0.02, **p = 0.002, Bonferroni‐corrected multiple comparisons. (B) Take lever responses during the 1‐h heroin relapse tests. #Two‐way ANOVA, food deprived vs. sated, *p* < 0.001; *p = 0.02, ***p = 0.0002, Bonferroni‐corrected multiple comparisons. (C) Locomotor activity test under sated conditions. Mean (±SEM) distance travelled (cm) in an open field for 1 h in rats with pPVT activated (J60) and control rats (vehicle).

Overall, acute food deprivation significantly increased heroin‐seeking (*Feeding condition: F*_(1, 16)_ = 14.26, *p* = 0.002, *η* ^*2*^ = 0.471; Figure 5A). Activation of the pPVT did not result in a statistically significant effect on seek lever responses (*Treatment*: *F*_(1, 16)_ = 4.466, *p* = 0.051, *η* ^*2*^ = 0.218), and the *Treatment x Feeding* interaction was not statistically significant (*F*_(1, 16)_ = 2.722, *p* = 0.118, *η* ^*2*^ = 0.145). The same pattern was observed for the number of take lever responses (*Feeding condition*: *F*_(1, 16)_ = 32.36, *p* < 0.0001, *η* ^*2*^ = 0.669; *Treatment*: *F*_(1, 16)_ = 2.756, *p* = 0.116, *η* ^*2*^ = 0.147; *Treatment x Feeding*: *F*_(1, 16)_ = 2.513, *p* = 0.132, *η* ^*2*^ = 0.136; Figure 5B). Bonferroni‐corrected multiple comparisons of seek lever responses revealed a statistically significant higher number of seek lever presses in the DREADD ligand (J60)‐treated rats compared to the vehicle‐treated rats under the food deprivation condition (*t*_(32)_ = 2.654, *p* = 0.025, Cohen's *d* = 0.938) and to the J60‐treated rats under sated condition (*t*_(16)_ = 3.837, *p* = 0.003, Cohen's *d* = 0.959). Multiple comparisons performed on the take lever presses identified statistically significant higher number of presses made by the J60‐treated rats under food deprivation condition compared to the sated condition (*t*_(16)_ = 5.143, *p* = 0.0003, Cohen's *d* = 1.286). A similar effect was observed in the vehicle‐treated rats (*t*_(16)_ = 2.901, *p* = 0.02, Cohen's *d* = 1.450).

There were no statistically significant differences in locomotor activity between the vehicle and J60‐treated groups in the open field (*t*_(16)_ = 1.416, *p* = 0.176, Cohen's *d* = 0.708).

### 3.3 Experiment 3: Effect of J60 Administration on Heroin‐Seeking in Rats Expressing mCherry

#### 3.3.1 Self‐Administration Training

**FIGURE 6:**
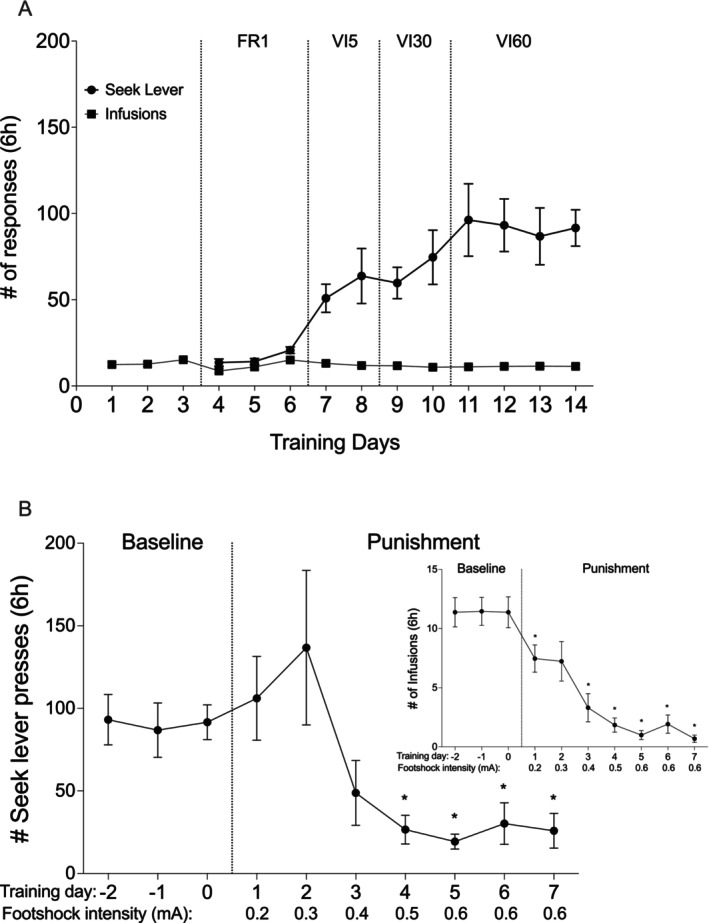
Experiment 3: mCherry control. (A) Heroin self‐administration. Mean (±SEM) of seek lever presses (circles) and number of heroin infusions (0.1 mg/kg/infusion; squares) over training days (*N* = 14). FR: fixed ratio, VI: variable interval schedule of reinforcement on the seek lever. (B) Punishment‐imposed abstinence. Mean (±SEM) of seek lever responses and infusions (insert) over punishment days. Baseline is the last 3 days of training under VI60. *p < 0.02 compared to last baseline day, Bonferroni‐corrected multiple comparisons.

Only males were used in this experiment. A total of six rats were removed from the analysis due to failure to acquire heroin self‐administration training or misplacement of the injector. Thus, 14 male rats were used for analysis. Rats demonstrated a statistically significant increase in seek lever presses over the training days (*Training days: F*_(2.482, 31.52)_ = 13.51, *p* < 0.0001, *η2* = 0.515) and a slight decrease in the number of heroin infusions over training days compared to the take lever only days (*Training days: F*_(4.967, 63.43)_ = 3.040, *p* = 0.016, *η* ^*2*^ = 0.192; Figure 6A).

#### 3.3.2 Punishment Phase

As the footshock intensity increased over days, rats significantly decreased heroin‐seeking (*Punishment days: F*_(2.153, 27.77)_ = 6.027, *p* = 0.006, *η* ^*2*^ = 0.318) and heroin‐taking (*Punishment days: F*_(3.172, 40.91)_ = 29.57, *p* < 0.0001, *η* ^*2*^ = 0.696; Figure 6B). Bonferroni‐corrected multiple comparisons revealed a statistically significant lower number of infusions beginning at the 0.2 mA day (with the exception of the 0.3 mA day) and a lower number of seek lever presses beginning at the 0.5 mA day compared to the last baseline day.

#### 3.3.3 Heroin‐Seeking Test

**FIGURE 7:**
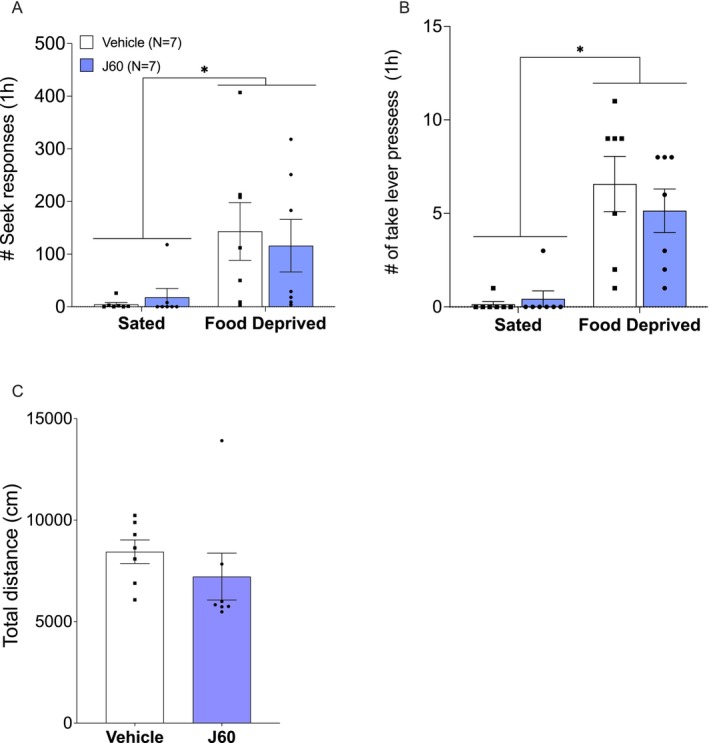
Experiment 3: mCherry control. Food deprivation‐induced heroin‐seeking tests in male rats. Rats transduced with AAV8‐hSyn‐mCherry were injected systemically (i.p.) with JHU37160 (J60; 0.1 mg/kg) or vehicle, 15–20 min before the relapse test. Mean (±SEM) of seek lever responses (A) and take lever responses (B) during the 1‐h heroin relapse tests under food deprivation (FD) and sated conditions. *Two‐way ANOVA, food deprive vs. sated, *p* < 0.05. (C) Locomotor activity test under sated conditions. Mean (±SEM) distance travelled (cm) in an open field for 1 h in rats with pPVT activated (‘J60’) and control rats (‘Vehicle’).

Food deprivation significantly increased heroin‐seeking and take lever responses in both the vehicle and J60 groups (*Feeding: F*_(1, 12)_ = 13.13, *p* = 0.004, *η* ^*2*^ = 0.522; *F*_(1, 12)_ = 38.59, *p* < 0.0001, *η* ^*2*^ = 0.763, respectively; Figure 7A,B). Administration of J60 had no effect on the heroin‐seeking (*Treatment: F*_(1, 12)_ = 0.026, *p* = 0.875, *η* ^*2*^ = 0.002) or number of take lever responses (*Treatment: F*_(1, 12)_ = 0.306, *p* = 0.590, *η* ^*2*^ = 0.025). The *Feeding x Treatment* interaction was not significant for either heroin‐seeking (*F*_(1, 12)_ = 0.386, *p* = 0.546, *η* ^*2*^ = 0.031) or number of take lever responses (*F*_(1, 12)_ = 0.913, *p* = 0.358, *η* ^*2*^ = 0.070). Administration of J60 had no statistically significant effect on locomotor activity (*t*_(12)_ = 0.947, *p* = 0.362, Cohen's *d* = 0.506, Figure 7C).

### 3.4 Histology—Experiments 1, 2 and 3

**FIGURE 8:**
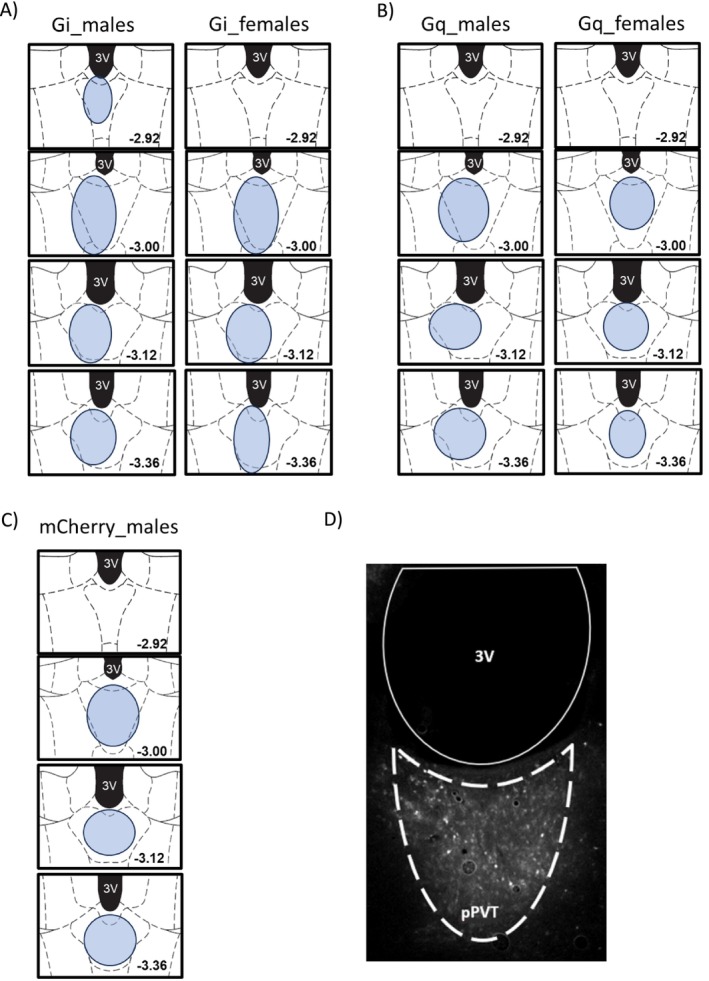
DREADDs expression in the pPVT. The extent of expression of hM4D(Gi)‐mCherry and hM4D(Gq)‐mCherry in the pPVT for Experiment 1 (A) and 2 (B), respectively. (C) mCherry immunofluorescence in the PVT for Experiment 3. (D) A representative sample of hM3D(Gi)‐mCherry immunofluorescence in the pPVT from Experiment 1. Distance from bregma: −3.00. 3V: third ventricle.

The extent of expression of hM4D(Gi) and hM4D(Gq) in the pPVT from Exp 1, 2 and 3 is presented in Figure 8A–C, respectively.

### 3.5 Experiment 4. In Vivo Electrophysiology Validation of Excitatory DREADDs

**FIGURE 9:**
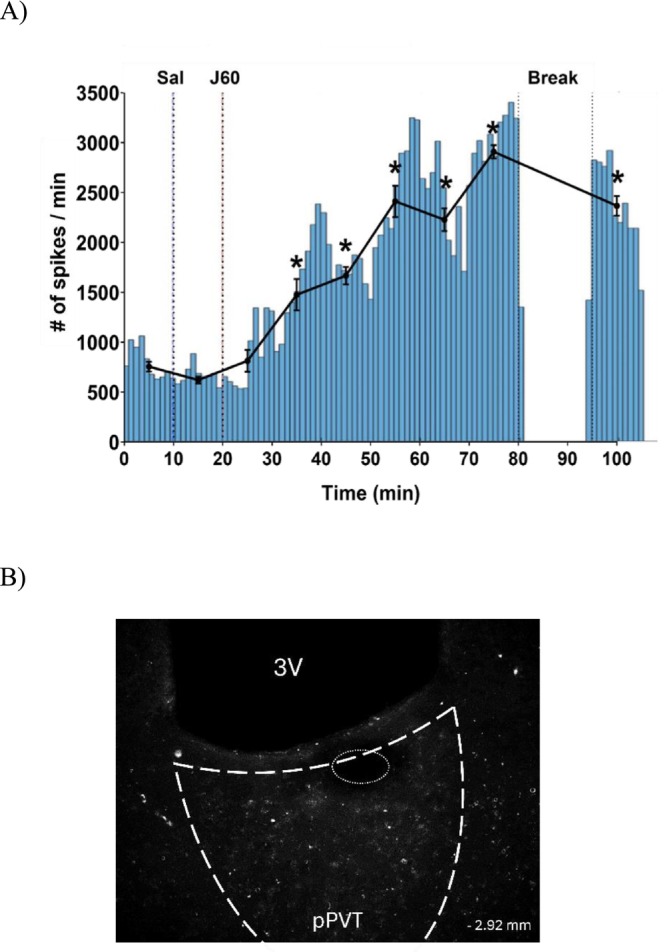
Single‐cell electrophysiology. (A) Histogram of the number of spikes per minute over the recording time and mean (±SEM) of the number of spikes per 10‐min bin (black line) from one neuron. *p < 0.001 compared to the mean number of spikes after saline (Sal) injection. Blue vertical line: saline injection; orange vertical line: J60 injection. (B) A representative sample of hM3D(Gq)‐mCherry immunofluorescence in the PVT from Experiment 4; the ellipse represents the post‐recording electrolytic lesion. Distance from bregma: −2.92. 3V: third ventricle.

To validate the use of J60 with excitatory DREADDs in the pPVT, we show in vivo electrophysiological recordings from one rat under anaesthesia before and after J60 injection (0.1 mg/kg, i.p.). Administration of J60 significantly increased single‐unit neuronal activity in the pPVT over 1 h and 30 min of recording (*Time: F*_(8, 81)_ = 63.05, *p* < 0.0001, *η* ^*2*^ = 0.862: Figure 9A). Saline injection (i.p.) was given 10 min after the baseline recording, which yielded no effect on neuronal activation (baseline vs. saline: *p* = 0.974), thus the post hoc analysis compared the mean spike rate of each time point with the mean spike rate under saline. The significant increase in the spike rate started 15 to 20 min after J60 injection (saline vs. 20 min after J60: *p* < 0.0001). At 1 h after the J60 injection, neuronal activity was still significantly higher than after saline injection (*p* < 0.0001). Neuronal activity was still significantly higher than saline even after 1 h and 20 min (saline vs. 1 h and 20 min after J60: *p* < 0.0001). A representative section of mCherry‐tagged hM4D(Gq) immunofluorescence with the electrolytic lesion is shown in Figure 9B.

## 4 Discussion

We investigated the role of the pPVT in food deprivation‐induced relapse to heroin‐seeking after punishment‐imposed abstinence. As expected, acute food deprivation robustly increased heroin‐seeking during the relapse tests. Inactivation of the pPVT significantly augmented food deprivation‐induced heroin‐seeking, an effect that was observed only in male rats. Chemogenetic activation of pPVT neurons also increased heroin‐seeking.

The augmentation of food deprivation‐induced heroin‐seeking by the inhibition of the pPVT is not very surprising. Inhibition of the pPVT facilitates reward consumption [15, 44, 45] and reward‐seeking motivated by cues [46, 47]. Paniccia et al. [33] recently reported that reinstatement of heroin‐seeking triggered by drug priming, cue or pharmacological stressor was associated with a selective inhibition of PVT‐NAc pathway neuronal ensembles. Other reports suggest that the pPVT‐NAc pathway supports behavioural inhibition. For instance, PVT‐NAc pathway neurons show an inhibitory response to reward‐predicting cues [48], and activation of the PVT‐NAc pathway underlies the suppression of sucrose‐seeking by behavioural suppressors (exposures to predator odour, pharmacological stressor or extinction; [49]).

In contrast, we have previously reported that chemogenetic inhibition of the pPVT did not affect heroin‐seeking after forced abstinence [36]. This discrepancy could be the result of the different abstinence procedure used in the current study, i.e., punishment‐imposed abstinence. The inclusion of a contingent punishment to heroin‐seeking leads to the development of lever aversion that does not exist in the forced abstinence procedure. A role for the pPVT in aversion responses is suggested by the innervation of the bed nucleus of stria terminalis and the central amygdala by pPVT neurons [50]. A functional role for pPVT in aversion was demonstrated by the suppression of the aversive symptoms of opioid withdrawal by silencing the projections from the pPVT to NAc [51, 52]. We suggest that inhibition of the pPVT could interfere with the aversion that developed towards the seek lever. A decrease in aversion towards the lever would allow for an increase in responses.

An alternative, but not mutually exclusive explanation for the impact of pPVT inhibition on behaviour is the potential role of the PVT in the attribution of the incentive value of reward cues. Inhibition of the PVT augmented cue‐induced reinstatement of cocaine‐seeking, albeit only in ‘goal tracker’ rats, i.e., those that attribute predictive, but not incentive, value to reward‐associated cues [47]. The authors suggested that inhibition of the PVT removed the suppression on the learned incentive motivational value of the cocaine‐associated cues in goal‐tracker rats [47]. Thus, the increase in heroin‐seeking following pPVT inhibition could be driven by disinhibition of behaviour and/or through an increase in the incentive value of heroin‐associated cues.

Notably, the pPVT manipulations augmented heroin‐seeking only under the food deprivation condition. A possible explanation taps into the role of the PVT in the resolution of motivational conflicts. Although we have not quantified it, we have observed a clear species‐typical conflict behaviour in the early days of the punishment phase (stretching towards the seek lever and reaching with a paw without touching, followed by a quick retreat; [53, 54]). McNally [55] described such phases as periods of instability, where behaviour oscillates between two competing demands (metastable behaviour). However, at the end of the punishment phase, rats rarely approached the seek lever, and the conflict behaviour disappeared. At that point, the behaviour became bistable, or in other words, the conflict had been resolved, and avoidance becomes the dominant behaviour [55]. It has been suggested that the PVT plays a critical role in bistable selection under motivational conflict conditions [55]. To achieve such a dynamic selection, the PVT is sensitive to changes in internal state, including caloric deficits. Indeed, the PVT receives input from several hypothalamic and brain stem nuclei that are involved in foraging and feeding [56]. For example, the PVT receives considerable inhibitory innervation from the neurons in the zona incerta that show increased activation under food deprivation conditions [57]. In addition, acute food deprivation (overnight fasting) was reported to decrease the neuronal activity of the pPVT in male and female mice [58]. Therefore, we suggest that the cumulative inhibitory inputs from the hypothalamus (driven by food deprivation) and the chemogenetic inhibition of the pPVT caused a stronger bias towards approach and lever press, and reduced aversion.

An interesting finding in our study is that *activation* of the pPVT did not attenuate the effect of food deprivation on relapse, as expected; rather, it resulted in an increase in heroin‐seeking. The observed increase was quite surprising considering the discussion above on the inhibitory function of the pPVT and its role in aversive behaviour. However, other studies support a role for pPVT activation in relapse to drug‐seeking. For example, optogenetic activation of the pPVT‐NAc pathway augmented heroin‐seeking following forced abstinence [35]. In addition, reinstatement of extinguished cocaine‐seeking was observed following intra‐pPVT infusions of Orexin‐A/Hypocretin‐1, which stimulate pPVT neuronal activity through local glutamate release [59, 60]. There is also indirect evidence that activation of the PVT underlies relapse to drug‐seeking. Lesions or transient silencing of the pPVT attenuated cocaine and alcohol‐seeking [28, 30, 31]. In addition, context‐induced relapse after punishment‐imposed abstinence was associated with activation of pPVT neurons [61].

There were no statistically significant sex differences during self‐administration and punishment phases. Yet, in both inhibition and excitation experiments, we observed a slightly faster punishment‐imposed inhibition of heroin‐seeking in the female rats. Inhibition of the pPVT only yielded a clear effect on food deprivation‐induced heroin‐seeking in male rats. However, the interpretation of the sex‐specific effects should be considered with caution because of the small number of female subjects and the lack of a statistically significant effect for sex in the omnibus analyses. Sex differences have been noted before for responses under conflict situations, as well as the involvement of the PVT in stress responses, although such data are still scarce. For example, female rats show higher tendencies for avoidance or risk aversion under motivational conflict [62, 63]; yet, we found no obvious differences in relapse between vehicle‐treated male and female rats under both feeding conditions. In addition, female mice demonstrated a more pronounced inhibition of pPVT neurons compared to males following an overnight fasting [58]. Thus, it can be argued that the chemogenetic inhibition of the pPVT in female rats could have no further impact on behaviour in an already inhibited pPVT.

The discussion on the functional role of the pPVT is further complicated by sometimes conflicting findings from studies that involve manipulation of the PVT with no discrimination between pPVT and aPVT, or nonselective manipulations of pPVT neurons. For example, Paniccia et al. [33] observed no effect on cue‐, drug‐ or stress‐induced reinstatement of heroin‐seeking in mice after a non‐specific chemogenetic activating the pPVT‐NAcShell pathway. However, selective opto‐stimulation of the pPVT projection neurons that innervate NAcShell parvalbumin (PV) interneurons (IN), combined with chemogenetic enhancement of the NAc^PV‐IN^ excitability, resulted in a *reduction* in stress‐induced heroin‐seeking. Moreover, the pPVT is composed of different populations, including *Estr1*, *Col12*, *galanin and Drd2‐*expressing neurons [64, 65]. It was recently reported that pPVT^D2+^ neuron projections to the NAcShell initiate food‐seeking, while pPVT^D2−^ neuron projections to NAcShell terminate food‐seeking [66]. To gain a better understanding of the role of the pPVT in stress‐induced relapse following voluntary abstinence, future studies would need to target specific output pathways (e.g., to the NAc or amygdala) and critical inputs (e.g., mPFC and hypothalamus) to the pPVT, as well as specific subpopulations in the pPVT and NAc.

In conclusion, our findings support the suggested role for the pPVT in relapse to heroin‐seeking, particularly following a challenge and when a motivational conflict requires resolution. The similar augmenting effect for inhibition and activation of the pPVT is intriguing and will require further investigation. Future studies should also focus on stress‐induced changes in the pPVT after punishment‐imposed abstinence by mapping neuronal activation in specific neuronal subpopulations. Finally, we were unable to further explore the sex‐specific effects of pPVT manipulations because of insufficient statistical power.

## Author Contributions

C.B. and U.S. designed research; C.B., A.D., A.M.‐B., and E.A.‐Y. performed research; M.D. and R.C. designed and performed electrophysiology experiment; C.B. and U.S. analyzed data; C.B. and U.S. wrote and edited the manuscript.

## Funding

This study was supported by the Natural Sciences and Engineering Council, Discovery Program (NSERC; RGPIN‐2016‐06694, RGPIN‐2023‐04731) and the Canadian Institute of Health Research (CIHR; PJT‐186231).

## Ethics Statement

All procedures were approved by the Concordia University Animal Research Ethics Committee and followed Canadian Council on Animal Care guidelines.

## Conflicts of Interest

The authors declare no conflicts of interest.

## Supporting information


**Data S1:** Supporting information.

## Data Availability

The data that support the findings of this study are available from the corresponding author upon reasonable request.
