# Supplementary material for: The Role of the Posterior Paraventricular Nucleus of the Thalamus in Food Deprivation‐Induced Heroin‐Seeking Relapse, in Male and Female Rats
Source: Addict Biol. 2026 Jan 8;31(1):e70115. doi: 10.1111/adb.70115 (PMC12782777; doi:10.1111/adb.70115)
Supplement: Supplementary file 1 — Data S1: Supporting information. [file ADB-31-e70115-s001.docx]

**The role of the posterior paraventricular nucleus of the thalamus in food-deprivation-induced heroin-seeking relapse, in male and female rats**

**Supplemental material**

**Materials and methods**

Subjects

Male and female Long Evans rats (Charles River, St-Constant, QC, Canada) weighing 250-300 g (2-2.5 months old) on arrival were used. The rats were maintained under a reversed 12 h light/dark cycle (9:30 AM light off) with ad libitum access to chow (Teklad 2018C, Inotiv) and water. Rats were pair-housed in a standard cage before surgery and then individually housed in the operant training chambers. All experiments were performed in accordance with the Animal Research Ethics Committee of Concordia University and carried out in accordance with the recommendations of the Canadian Council on Animal Care.

## Apparatus

## Experiments were performed in standard operant conditioning chambers (Coulbourn Instruments, Allentown, PA, USA; 29.0 cm × 29.0 cm × 25.5 cm) that were individually located in sound attenuating boxes. Each chamber was equipped with two retractable levers on the same wall (9 cm above the grid-floor), a house light, white cue lights above the levers, and a tone generator (2.9 kHz, Sonalert, Coulbourn Instruments). An infusion pump was connected to the catheter port via a liquid swivel (Lomir Biomedical, QC, Canada) and Tygon tubing shielded with a metal spring.

Intravenous and Intracranial Surgeries

Intravenous catheters were implanted into the right jugular vein under 2% isoflurane anesthesia as previously described (Chisholm et al., 2021). During the same surgery, after catheter implantation, rats were injected with 0.6 μl of viral vector (Canadian Neurophotonic Platform/viral vector team, QC) into the pPVT: −3.0 AP, 1.15 ML, −5.6 DV relative to Bregma, at a 12° angle. Viral vectors were injected at a rate of 0.1 μl/min and left in place for an additional 10 mins. The following viral vectors (Canadian Neurophotonic Platform/Viral

Vector Core, Québec, QC) were injected: *Experiment 1*. AAV8‐hSyn‐hM4D(Gi)‐mCherry; *Experiment 2*. AAV8‐hSyn‐hM3D(Gq)‐mCherry; *Experiment 3.* AAV8-hSyn-mCherry; *Experiment 4.* AAV8‐hSyn‐hM3D(Gq)‐mCherry, without catheterization. Before surgery rats were administered 0.9% saline (2.0 ml, s.c.) and the analgesic Ketoprofen (5.0 mg/kg, s.c., CDMV Inc., St-Hyacinthe, QC). Post-op care included administration of 0.9% saline and Ketoprofen immediately after surgery, and for the following two days, once a day.

Drugs

## Heroin HCl (provided by the National Institute for Drug Abuse, Research Triangle Park, NC, USA) was dissolved in 0.9% sterile saline. Clozapine-N-oxide (CNO, provided by National Institute for Drug Abuse, Research Triangle Park, NC, USA or Cayman Chemical, Ann Arbor, MI, USA) was used as the ligand to activate the DREADDs for half of the male rats in Experiment 1. CNO was dissolved in 5% dimethyl sulfoxide (DMSO) and 95% sterile saline. A 5% DMSO solution in 95% sterile saline was administered as vehicle control. For the other half of rats in Experiment 1, JHU37160 (J60, HelloBio, Princeton, NJ, USA) was dissolved in saline and used as the ligand to activate the DREADDs. Sterile saline was administered as vehicle control.

Procedures

A timeline for all experiments is provided in **Figure 1.**

*Heroin self-administration*: Rats were trained to self-administer heroin for 6 h on daily sessions under a seeking-taking chain schedule of reinforcement, as previously described (Borges et al., 2022). Briefly, for the first 3 days of training, rats had access only to the take lever at FR1. In the following days, we introduced the seek lever to initiate the training on the seek-take chain schedule. Each seeking-taking cycle began with the illumination of the house light and the insertion of the seek lever, with the take lever retracted. The first press on the seek lever initiated a variable interval (VI) schedule, and the first lever press after the expiry of the VI resulted in the retraction of the seek lever and insertion of the take lever. Pressing the take lever (fixed ratio 1, FR1) resulted in retraction of the lever, extinction of the house light, administration of one heroin infusion (0.1 mg/kg/infusion; 0.13 ml over 5 s), and presentation of the cue light and the tone for 20 s. The programmed schedule of reinforcement for the seek lever advanced over training as follows: starting at FR1 for the first 4 days of training, followed by VI5 for 2 to 3 days, then VI30 for 3 days and VI60 for the last 3 days. The inter-trial interval (ITI) progressively increased over sessions from 30 s to 5 min. If the rat failed to complete the seeking-taking chain after 10 min, the trial ended, and the ITI was initiated.

*Punishment*: During the punishment phase, 30% of the completed seeking links were punished with a 0.5 s footshock delivered through the chamber grid-floor, instead of insertion of the take lever. The remaining 70% of the completed seeking links were identical to self-administration training. The ITI on both punished and non-punished trials was 5 min. Rats went through 6 days of punishment-imposed abstinence in which the footshocks intensity gradually increased by 0.1 mA over days, from 0.2 mA to 0.6 mA (the last 2 days on 0.6 mA). In Experiment 1, male rats received an exploratory treatment during punishment (CNO, 6 mg/kg, i.p; or J60, 0.1 mg/kg, i.p.) or Vehicle (1.0 ml/kg, i.p.) to assess the effect of pPVT inhibition on behavior under punishment. No behavioural effects were observed, and data are not reported here.

*Food deprivation-induced heroin seeking tests*: Following the punishment phase, rats underwent two counterbalanced 1-h relapse tests, 48 h apart, under the seeking-taking VI60 schedule with an ITI of 5 min in the presence of the drug-associated cues (light and tone). Neither heroin nor footshock were delivered. A DREADD ligand (J60: 0.1 mg/kg or CNO: 6 mg/kg, i.p.) or Vehicle (1.0 ml/kg, i.p.) was injected 20 mins prior to the beginning of each test. No significant differences in behavior were observed for the two DREADD ligands. Rats were randomly reassigned into the DREADD activation (Experiment 1: inhibitory DREADDs; Experiment 2: excitatory DREADDs) or control groups. For the food deprivation condition, rats had no access to food for the 24 h preceding and during the test session. During the sated condition (Sated), rats had unlimited access to food. Water was available *ad libitum* to both groups. Food was returned to the food-deprived rats immediately after the test session. The next morning, food was removed from the previously sated rats, and the second relapse test was conducted 24 h later.

*Locomotor activity*: The day after the last relapse tests, rats were injected with either a DREADD ligand (J60: 0.1 mg/kg, i.p. or CNO: 6 mg/kg, i.p.) or Vehicle (1 ml/kg, i.p.) 20 mins prior to being placed into a locomotor activity monitoring chamber (Coulbourn Instruments). Total distance travelled (m) was recorded during a period of 1 h using the TruScan software (Coulbourn Instruments).

*Electrophysiological validation of excitatory DREADD activation in the pPVT*: One Long Evans rat was infused with AAV8‐hSyn‐hM3D(Gq)‐mCherry in the pPVT, as described in the “Intravenous and Intracranial Surgeries” section. After 8 weeks, neuronal activity was recorded *in vivo*, under isoflurane anaesthesia, before and after J60 injection (0.1 mg/kg, i.p.), using saline injection (i.p.) as control. Methods for unit recordings closely followed published procedures (Lévesque et al., 2020). Using multiple single electrode headstages, held onto the digital stereotaxic instrument, the recording location target was at −3.0 AP, 0 ML, −5.4 DV relative to Bregma, aiming for the pPVT. One bone screw served as the ground contact and a stainless-steel wire placed in brain tissue served as the reference. Three to four tungsten microelectrodes with shank diameters of 75 μM and impedances around 1 MΩ (0.2-1.5 MΩ – FHC Inc., Bowdoin, ME, USA) were inserted into individual drives, mounted onto the headstage. Each microelectrode could be moved independently using a small screwdriver to advance and retract the electrode. For unit activity, the signal was captured as a digitized wide-band signal (0.1-6000 Hz) and as digitized action potential waveforms filtered between 600 and 6000 Hz, both sampled at 32 kHz. The wide-band signal was then filtered according to LFP (0.1-50 Hz) and multi-unit (600-6000 Hz) bands. Spike isolation was achieved by lowering or retracting the individual microdrives (250 μm/turn precision), with usual increments of about ¼ or ½ turn. Adjustments on spike detection were then performed on the Neuralynx DAS software 32-point digitized thresholded waveform, overlaid to verify reproducibility. In addition, the wide-band unit signal was played on a loudspeaker sound output to monitor waveform stability, useful during microelectrode positioning. At the end of the recording sessions (approximately 120 min), an electrolytic lesion (100 μA, 35 s, anodal) was made where single units were found, while the rat was still under anesthesia.

*Histology*: Following the locomotor activity test or electrophysiological recordings, rats were transcardially perfused with PBS followed by 4% paraformaldehyde. Brains were extracted and postfixed for 24 h, cryoprotected with 30% sucrose at 4°C for 48 h, stored in −80°C, and coronal sections (40 μm) were sliced on a cryostat. DREADD expression and the electrolytic lesion were determined under a fluorescent microscope with reference to a brain atlas (Paxinos & Watson, 2005).

Statistical Analyses

*Behaviour.* Statistical analyses were conducted using GraphPad Prism 9 or 10.2.3 (Boston, MA, USA). For self-administration training data, we used two separate two-way mixed ANOVAs or mixed-effect analyses to compare the number of seeking responses and infusions between males and females over the self-administration days (*Sex* as between-subject factor and *Training day* as within-subject factor). Behaviour during the punishment period was analyzed using mixed-effect ANOVA (*Sex* as a between-subject factor and *Punishment days* as a within-subject factor). Geisser-Greenhouse’s epsilon correction was used when sphericity could not be assumed. Because our design was not powered for comparing performance in the relapse tests between sexes, seek and take lever responses tests were analyzed using 2-way ANOVAs, with *Treatment* (J60, Vehicle) as the between-subject factor and *feeding condition* (Sated, Food Deprivation) as the within-subject factor, collapsed over sex. Specific multiple comparisons were done using Bonferroni corrections. Locomotor activity data (distance travelled) were analyzed with an unpaired t-test comparing J60 and Vehicle groups. The effect sizes reported are Cohen’s coefficient (*d*) and partial eta squared (*ƞ^2^*). Statistical significance was defined as *p* ≤ .05.

*In vivo electrophysiology data analysis.* Multi-unit and single-unit quantitative analyses were performed using NeuroExplorer (Nex Technologies, Littleton MA) and MATLAB (MathWorks, Natick, MA), the latter with routines based on standardized functions (e.g., signal processing toolbox). For unit data, the digitized spikes were processed for single-unit identification. For single electrode recordings, this was done in SpikeSort (Neuralynx, Bozeman, MT, USA) in manual mode, focusing on spikes corresponding to an adapted extracellular action potential shape template, estimated from the overlaid spikes (based on > 100 detections). Secondary elements were also used such as the 2-D scatterplot distribution of the spike amplitude and duration values (e.g., peak height, valley depth, action potential duration). Interspike intervals (ISIs) of less than 1 ms were removed. Generally, units with fewer than 2% of the ISIs under 3 ms were kept. We produced histograms and binned the data (1-min bins, rate of spikes/min) around the injections to assess change throughout the experiments. These bins were then converted into 10-min aggregate epochs for the duration of the experiment. We used GraphPad Prism to run a one-way ANOVA on the firing rate data over time (in the example on Figure 9, throughout the 1h30 of recording).
